# Language interpretation conditions and boundaries in multilingual and multicultural emergency healthcare

**DOI:** 10.1186/s12914-018-0157-3

**Published:** 2018-06-05

**Authors:** Christina Lundin, Emina Hadziabdic, Katarina Hjelm

**Affiliations:** 1 Department of Social and Welfare Studies, University of Linköping, Campus Norrköping, S- 601 74 Norrköping, Sweden; 2 Department of Health and Caring Sciences, Faculty of Health and Life Sciences, Linnaeus University, Växjö, Sweden; 3 Department of Public Health and Caring Sciences, Uppsala university, Uppsala, Sweden

**Keywords:** Emergency care, Health care professionals, Language interpreter practices, Migrants’ health, Organization

## Abstract

**Background:**

With an increasing migrant population globally the need to organize interpreting service arises in emergency healthcare to deliver equitable high-quality care. The aims of this study were to describe interpretation practices in multilingual emergency health service institutions and to explore the impact of the organizational and institutional context and possible consequences of different approaches to interpretation. No previous studies on these issues in multilingual emergency care have been found.

**Methods:**

A qualitative descriptive study was used. Forty-six healthcare professionals were purposively recruited from different organizational levels in ambulance service and psychiatric and somatic emergency care units. Data were collected between December 2014 and April 2015 through focus-group and individual interviews, and analyzed by qualitative content analysis.

**Results:**

Organization of interpreters was based on patients’ health status, context of emergency care, and access to interpreter service. Differences existed between workplaces regarding the use of interpreters: in somatic emergency care bilingual healthcare staff and family members were used to a limited extent; in psychiatric emergency care the norm was to use professional interpreters on the spot; and in ambulance service persons available at the time, e.g. family and friends were used. Similarities were found in: procuring a professional interpreter, mainly based on informal workplace routines, sometimes on formal guidelines and national laws, but knowledge of existing laws was limited; the ideal was a linguistically competent interpreter with a professional attitude, and organizational aspects such as appropriate time, technical and social environment; and wishes for development of better procedures for prompt access to professional interpreters at the workplace, regardless of organizational context, and education of interpreters and users.

**Conclusion:**

Use of interpreters was determined by health professionals, based on the patients’ health status, striving to deliver as fast and individualized care as possible based on humanistic values. Defects in organizational routines need to be rectified and transcultural awareness is needed to achieve the aim of person-centered and equal healthcare. Clear formal guidelines for the use of interpreters in emergency healthcare need to be developed and it is important to fulfill health professionals’ wishes for future development of prompt access to interpreters and education of interpreters and users.

## Background

Globally, the migrant population has increased in recent years, resulting in an increasing number of foreign-born persons not speaking the official language of the host country [1]. Language barriers have significant impact in healthcare for both the patient and the system and may lead to health inequalities [2], patient safety risks [3], increased costs related to misdiagnosis and repeated visits, and ineffective use of resources [4], limited access to diagnoses, diagnostic testing, and treatment [5, 6], longer stays at the emergency department [7, 8], and migrant patients may receive fewer explanations and follow-ups [6].

To overcome language barriers and improve healthcare and satisfaction for people who do not speak the official language, the use of professional interpreters is recommended in previous literature review studies [9, 10]. In Sweden all persons in contact with public authorities who need it have the right to an interpreter according to the Management Act [11] in order to prevent disparities and to deliver equal care based on the individual’s active participation.

Previous studies in primary healthcare investigating adverse events in the use of professional interpreters showed that the main problems were related to organizational issues and the interpreters’ limited language competence [12]. Besides our previous study focusing on use of interpreters in multilingual older persons [13], no investigations have been found in the literature review concerning organizational issues in emergency care.

The main results of the recent study [13] showed that in elderly care, interpreting practice was closely linked to institutional, interpersonal, and individual levels, and guidelines for arranging the use of interpreters at workplaces were lacking on different levels in the organization. Professional interpreters were used on predictable occasions planned long in advance during office hours and mainly in consultations with physicians or for individual care planning. In everyday care situations and on unpredictable occasions bilingual healthcare staff and family members were used. Health professionals also expressed a need for translation of written documents such as menus, test results, etc.

The use of professional interpreters was not adapted to the context of multilingual elderly healthcare [13]. However, it has been found that professional interpreters were underutilized in emergency healthcare [14, 15] and the question is whether different problems/strategies are found in other health care contexts with different characteristics. Are there differences in high versus low structured activities or limited timeframe versus longstanding contacts between emergency care and elderly care? In emergency care, the interpreter practice and its organization is distinctive in that it usually occurs in less structured activities with high intensity and often with use of high-tech measures and delivered in a limited timeframe, in contrast to elderly healthcare where healthcare is delivered in less structured activities in longstanding contacts.

### Theoretical framework

This study is based on transcultural care [16], communication in institutions and organizations [17], and organizational routines [18, 19].

Communication is fundamental for healthcare professionals, as is cultural understanding [16, 20]. Routines impact the care of the patient, and social structure and the culture of an organization are dimensions which affect health professions and impact their actions [21, 22].

Transcultural care can be described as the need to provide care based on a person’s or group’s cultural beliefs, values, and practices in order to promote or regain health, and emphasizes that communication is fundamental for transcultural caring [16]. Caring is also influenced by the social structure and the cultural context existing in an organization [16, 21]. Health professionals’ routines impact patients’ health [22] and fundamental dimensions of nursing such as humanism (social, educational, ethical and spiritual dimensions) and its antithesis (elements of bureaucracy, economic, political, legal, and technological dimensions) impact nursing actions [21]. To describe identification with a certain custom or specific country [16] the power relation between migrant minorities and the majority, which defines specific individuals/groups as non-associated and others as related [23], the concept of ethnicity is used [16]. How healthcare professionals use and discuss language interpretation is also described as affecting the area of ethnicity at the workplace [24], and ethnicity can be understood as socially constructed boundaries between people with shared history or shared cultural values [25]. Thus, holistic and individualized healthcare recognizes human rights to health including individual autonomy and safety, such as having basic rights and freedom to access quality healthcare, and that individuals should be treated fairly, equally and impartially [26].

Communication can be described as a multifaceted phenomenon which makes a person’s identity visible and shows how the other person is perceived on the individual and the social level [27]. It also makes it possible for the individual to participate in society [17], where the individuals have legal and health literacy including a certain ability to understand, assess, access and communicate selected information about the care obtained, confronting social injustice either for themselves or their families in order to achieve health and well-being [28]. Increasing migrant health literacy enables migrants to have a better access to appropriate health care, which in turn promotes social justice by increasing migrants’ social engagement, inclusion and full citizenship. However, communication in an institutional context differs and can be asymmetrical when patients and healthcare staff do not have the same aims, knowledge, or resources, particularly as health professionals have their own agenda for assessment of information on which to base decisions and planning of care [17].

Organizational routines are progressive structures used to accomplish organizational work aimed to deliver high-quality care with equitable outcomes for different ethnic groups in society [16]. These can be defined as a repetitive, recognizable pattern of interdependent actions [29], involving five actors dynamically and equally operating with one another [18, 19]: (1) the individual, which includes the person’s identity, values, goals, and competence; (2) the interpersonal interaction including social skills, personality, power, and influence; (3) the organizational context comprising technological, cultural, and coordination structures; (4) the institutional context with regulative, normative, and cultural-cognitive pillars; and (5) the environmental context with economic/political climate, legislative constraints, demographic changes, and development of technology [18, 19] (for further details see [13]).Communication through an interpreter is thus a complex phenomenon [13].

## Method

### Aim

The aim of the study was to describe language interpretation practices in multilingual emergency health service institutions. The study explored the impact of the organizational and institutional context and possible consequences of different approaches to interpretation. The central research question of the study was: How do emergency care health professionals in Sweden influence culturally and linguistically diverse patients’ access to language interpreters?

### Design

A qualitative exploratory and descriptive study was used to capture healthcare professionals’ experiences in a field not previously explored and to describe their experiences in their own natural state [30]. Semi-structured interviews, individual and in focus-groups, with health care professionals working in emergency care institutions, were conducted to investigate and understand the reality of interpreter service when communicating with patients of different linguistic backgrounds. Semi-structured interviews allow informants to elaborate on experiences and thoughts, while at the same time staying within certain topics designed by the research team [30]. In focus-group interviews participants can open up through group interaction for discussion where different perspectives, even more or less unconscious, can be revealed [31].

### Setting

Emergency care institutions in somatic care departments (two emergency clinics (open 24 h) with observation wards for maximum 48 h’ care)) and psychiatric departments (two psychiatric emergency clinics (open 24 h), one including a psychiatric intensive care unit) and paramedics in ambulance care at two county hospitals in two different cities in migrant-dense regions in Sweden were studied. The cities had approximately 88,000 vs 137,000 inhabitants, of whom 16.7 vs 17.8% were foreign-born [32]. All settings are characterized by high intensity, with brief encounters between staff and patient and often delivered in a high-tech environment [33]. However, the encounters can differ from minutes up to days. Care in an emergency care clinic or observation unit is focused on assessment of an individual’s status and first-aid measures, triage and/or referral of the patient for further care [33]. Care delivered in the psychiatric clinic or psychiatric intensive care unit is mainly focused on acute measures connected to severe dysfunction of mood, behavior, perception, or thoughts that might be a threat to life, psychological integrity or adequate functioning. Care delivered in ambulance service is prehospital and limited to transportation to the hospital and life-sustaining measures.

Like the majority of health care institutions in Sweden, all the emergency care institutions studied are run by the county council with the exception of one of the ambulance service units that is run by a private enterprise, although with the same regulations. The professional interpreters they refer to are procured under the Public Procurement Act [34] to guarantee the quality of the interpretation service, and the interpreter agencies used by the hospitals are run by private enterprises outside the health care system. In the studied areas the healthcare and thus interpreting practice followed Swedish legislation: the Management Act [11], the Swedish Health and Medical Services Act [35], the Swedish Patient Act [36], and the Public Procurement Act [34]. For further details see [13].

### Participants and procedure

A purposive recruitment procedure was chosen to ensure variation [30] in professions, gender, and experiences of working with interpreting situations in emergency care for foreign-born persons of different linguistic backgrounds and on different levels in the organization. Respondents were recruited after information meetings at the workplace (*n* = 22) or by asking respondents during the interview period (snowball strategy, [13]) to invite those who had not attended the information meetings held before the study (*n* = 24).

Managers in the different emergency care institutions were contacted by telephone by the investigators (CL, EH) to give information about the study and get approval for the implementation. Verbal and written information about the study was given. After their approval, the managers were asked to invite health care staff who had experience of communication with non-Swedish-speaking persons in emergency care to participate. A time was set for information meetings at the workplaces where the respondents received verbal and written information about the study. Those interested in participating provided their contact details by e-mail or in written form to the investigators and were then contacted to set a time and choose an appropriate place for the interview.

The study population included 46 persons, 14 males and 32 females, aged 21 to 65 years (median 37), with occupational background as nurses, nurse assistants, nurse-paramedics, paramedics, physicians, and social workers and with work experience in emergency care from 1 to 28 years (median 4 years) (see Table 1). Eleven of the participants were in a leading position and working as managers and the rest as ordinary health care staff (employees).

**Table 1:** Characteristics of the study population

Variable	Persons *N* = 46
Gender (n)	
Female	32
Male	14
Age (years)^a^	37 (21–65)
Country of birth	
Sweden	41
Ex Yugoslavia	3
Finland	1
Iraq	1
Professional level (n)	
Nurse	21
Assistant nurse	8
Physician	5
Social worker	1
Unit managers	6
Operational manager	5
Emergency healthcare context (n)	
Ambulance care	8
Somatic emergency clinic	26
Psychiatric emergency clinic	12
Work experience in health care (years)^a^	13 (1–44)
Work experience in emergency healthcare (years)^a^	4 (1–28)

^a^Median (range)

### Data collection

Data were collected by semi-structured interviews, individually and in focus-groups, between December 2014 and April 2015. An interview guide was developed based on a previous study [13] and other investigations in the area [12, 37–39]. The questions in the interview guide focused on when interpretation became an issue, how interpretation was conceptualized, what was supposed to be interpreted and the implications of different interpretation practices and challenges in improving equal healthcare concerning interpreting service. The first focus group was used as a test of the interview guide [31] and led to minor changes by also asking about the need for a policy on interpreting.

The interviews were led by two nurses experienced in leading groups and qualitative studies in research on migrants and use of interpreters (first and second author). All interviews were held in secluded rooms chosen by the informants at their workplaces.

The interviews were conducted with 46 participants, in five focus-groups with 3–5 persons in each group (*n* = 20) and in 26 individual interviews. The focus-groups were, as recommended, planned to be homogenous in terms of profession to develop a comfortable group dynamic and avoid negative influence of power imbalance between different professions [31]. Three groups included only registered nurses vs assistant nurses vs. managers and there was one group with three assistant nurses and a registered nurse. The focus-group interaction was friendly, lively, and well-balanced in gender-mixed groups and the discussions lasted 60–70 min. In the individual interviews the communication proceeded without problems or interruptions in a free-flowing way and lasted 30–60 min. Directly after the interviews, the interviewer made notes on the content of the interviews, the group interaction, and feelings developed during the interview [31]. All interviews were audio-recorded and transcribed verbatim by a professional secretary and one of the investigators (CL), and then analyzed.

### Data analyses

Data were analyzed with inductive qualitative content analyses [30]. The transcripts were read through and checked for accuracy and coherency to promote high quality and to get a sense of the content as a whole [30]. Then, the texts were broken into smaller meaning units and codes were identified. Codes with similar meanings were grouped together and subcategories and categories emerged based on patterns. Throughout the analysis process the investigators searched for contradictions, regularities, similarities, and patterns in the text supporting the development of subcategories and categories. Categories were refined and developed until an acceptable system was reached. Collection and analysis of data proceeded concurrently and until no new information was added in the data analysis [30, 31].

In order to increase credibility, investigator triangulation was used, by two of the authors independently coding and checking the content of the codes [30] which showed high agreement. During the whole process of analysis all co-authors also checked and confirmed the content and the relevance of subcategories and categories. The authors have different experiences in research and practical work in healthcare but all are in research in the area of migrants’ health and using qualitative methods. Discussions were held until consensus was reached in the event of diverging results. By illustrating categories with illuminative quotations and by naming categories as close to the text as possible, confirmability was ensured. By describing the investigation process (audit trail), as thoroughly as possible, dependability was confirmed, and finally transferability can be strengthened by studying informants with different professions, from different hospitals and different geographical areas, thus giving a precise description of the study population.

## Results

The organization of interpreting for healthcare staff in emergency care institutions concerning encounters with persons with language barriers is described in four categories, with subcategories, summarized in (Table 2): 1) the use of interpreters is determined by the patient’s health status and access to interpreting service in the organization; 2) utilization of interpreting services is driven by informal or formal guidelines and different national laws; 3) the use of a professional interpreter at the workplace depends on the interpreter’s linguistic skills, personal qualities, professional approach, and organizational aspects; and 4) recommendations to improve the use of interpreting services in emergency care.

**Table 2:** Overview of categories with subcategories analyzed from interviews by healthcare staff working in multilingual emergency healthcare

Category (No. of statements)	Sub-category (No. of statements)
The use of interpreters is determined by the patient’s health status and access to interpreting service in the organization (424)	Type of emergency care determines the mode of interpreting (338) The patient’s health status and availability of an interpreter determine the type of interpreter used in the workplace (44) Healthcare staff in somatic and psychiatric emergency care prefer professional interpreters (24) Healthcare staff in the ambulance prefer family members on the spot (18)
Utilization of interpreting services is driven by informal or formal guidelines and different national laws (412)	Informal and formal guidelines and limited knowledge of existing laws govern utilization of interpreting services in the workplaces Interpreters are used in situations with communication deficiencies in somatic and psychiatric emergency care (136) Professional interpreters are not used in ambulance care or in urgent situations (16) Interpreters are not used if body language can be used for communication (12)
The use of a professional interpreter at the workplace depends on the interpreter’s linguistic skills, personal qualities, professional approach, and organizational aspects (290)	The professional interpreter’s linguistic competence, positive personal qualities, and professional approach facilitates work when communication is deficient (206) Professional interpreter perceived as positive and a tool facilitating communication (24) Professional interpreter perceived positively or negatively when organizational aspects (time, environment and technical equipment, and interpreter languages) of the use of interpreter work or not (18)
Recommendations to improve the use of interpreting services in emergency care (67)	Developing the procedure for prompt access to professional interpreters in the workplace (58) Education of health professionals in using a professional interpreter, and the professional interpreter’s role in various care situations (9)

### The use of interpreters is determined by the patient’s health status and access to interpreting service in the organization

#### Type of emergency care determines the mode of interpreting

A common habit in somatic and psychiatric emergency healthcare, as described by the respondents, was to use professional interpreters, while health professionals in ambulance care used family members.

*A professional interpreter in the room is hard to beat. It is the best … the patient often feels much more secure, the interpreter gets a different kind of contact with the patient and might then get more information just through his presence.* (Respondent=R29, Psychiatric emergency clinic (=Psychiatric EC))

*The advantage is that they* (family members) *are often on the spot. Sometimes they have contacted relatives by telephone and that works well too, as I described earlier with FaceTime that you just stand there talking and then pass the receiver between you. It is mostly relatives that we use*. (R 22, Ambulance care)

Differences between workplaces were found: bilingual health professionals and family members could be used to a limited extent in ambulance care and emergency care, but in the latter case telephone interpretation by professional translators was also used on unpredictable occasions for short-term assignments, even outside office hours. In some situations healthcare staff had a negative perception of having professional interpreters present, due to threatening patients in psychiatry, being exposed to unpleasant emergency situations, and limited space in ambulance care. In psychiatric emergency care, the norm was to have professional interpreters in the room related to characteristics of the patient’s mental health status, e.g. depression, and delusion with paranoia that might lead to suspiciousness of telephone interpretation and using family members.

*In psychiatry we need to have an interpreter present all the time. There are no caring situations where you think “we need to try to manage this without an interpreter.” It doesn’t work. We’re not mind readers.* (R 24, Psychiatric EC)

However, both somatic and psychiatric emergency healthcare found it a problem to have professional interpreters in the room because they were not easily accessible at short notice but required long planning in advance and in some cases led to prolonged healthcare visits for the patient.

*Often you have to book time, and sometimes there are no times available. Even if there is some sort of emergency line – if I have understood right – where you can get an interpreter within an hour or half an hour. And then I don’t know whether I can be in place.* (R 13, Somatic emergency clinic (= Somatic EC))

Most health professionals perceived benefits in using telephone interpretation in short-term, one-off emergency care situations, and in situations experienced as sensitive by patients.

*An interpreter by telephone is more easily accessible. Sometimes … the only alternative to get an interpreter in the language we needed … it might be possible to get one faster for the same reason.* (R 13, Somatic EC)

In some cases, using telephone interpreters had been experienced as a hindrance in examinations as they were unable to observe body language and the technical equipment functioned badly, making it difficult to hear.

Healthcare professionals could see advantages in using calm and neutral family members as interpreters in some healthcare situations when information transfer occurred concerning the patient’s healthcare status, especially in ambulance care when they usually do not know what language they will meet. The patient feels security and trust in the family member, who is mostly available in unpredictable situations at short notice. At the same time healthcare staff can easily convey information to the relatives. Disadvantages could be poorer-quality interpretation because of risk of breaking the code of confidentiality and lack of language competence.

Using bilingual healthcare professionals was mainly felt to be a good choice as they were “easily accessible and already in place.” However, they could cause problems by not being neutral when interpreting and “not keeping the code of confidentiality,” and having limited language knowledge.

*… can see advantages but it is a person … who is used to health care and … patient contact and knows the language at the same time … as long as they don’t have a personal relationship to the family…* (R 9, Somatic EC)

#### The patient’s health status and availability of an interpreter determine the type of interpreter used in the workplace

Respondents described how the patient’s health status affected the choice of type of interpreter and vice versa. Informal interpreters such as relatives or colleagues were used when necessary if the patient was unconscious, severely ill, unable to speak, or action had to be taken without delay, or in the case of a heavy workload and need of fast access to an interpreter.

To help the patient receive information quickly and thus save time, sometimes bilingual colleagues were used as interpreters to assess the patient’s health problems for referral to the right level or type of care. Diffuse conditions, complex care, and critical conditions require interpreters, in contrast to simple health problems.

*If it’s severely ill patients, critically ill, then the interpreter actually doesn’t matter so much, because in those situations we have guidelines that … so we have to … drive to the nearest hospital as soon as possible and then there’s no time and it doesn’t matter…* (R 15, Ambulance care)

*… if there’s someone consulting for … a minor complaint or if you have to decide whether they should go to another caregiver … then it’s faster to go and get a colleague.* (R 5, Somatic EC)

#### Healthcare staff in somatic and psychiatric emergency care prefer professional interpreters

In somatic and psychiatric care situations health professionals preferred a professional interpreter in place because they perceived them to ensure interpreting word-by-word, having a professional attitude, and remaining neutral and objective.

*A professional interpreter on the spot … is the best. That it should not be colored by relatives, relatives can influence the conversation and you rely on a professional interpreter to actually interpret … That’s what one is looking for…* (R 27, Psychiatric EC)

#### Healthcare staff in the ambulance prefer family members on the spot

In situations in the ambulance with no other choice of interpreting service, in situations that occur in the home or various social environments, the staff in ambulance services prefer a calm, objective relative who is on the spot. This is because they can give security and can be an asset in a confused situation, also providing continuous information about the person.

*… sometimes it can be … a great advantage that there can be relatives who know their father… Relatives have a different knowledge about the person … they can help on another level also with such things that often help us and that don’t concern language … it has a dual function.* (R 20, Ambulance care)

### Utilization of interpreting services is driven by informal or formal guidelines and different national laws

#### Informal and formal guidelines and limited knowledge of existing laws govern the utilization of interpreting services

Knowledge about laws, policies, and guidelines regarding interpreting service varies among health professionals, and the majority are not aware of any specific law or policy regulating the use. Most health professionals’ decisions regarding professional interpreter use depend to a great extent on what they consider to be the patient’s needs, and from the perspective that the person has a right to an interpreter to understand information given. Most of the health professionals know about the law on procurement governing routines for booking a professional interpreter.

R 1:4: *No, not using an interpreter … no.*

R 1:3: *I was thinking of the law on patient safety, isn’t there such a law? Aren’t interpreters included in that?*

R1:2: *They* (patients) *have the right to, the Health and Medical Care Act,…*

R1:4 *… right to the same care and but not to an interpreter, no.*

R1:3*: But they* (patients) *know that they have the right to an interpreter …*

R1:2 *… but then whether it is regulated in law, on which occasions, to what extent, I can’t say.* (Somatic EC)

In psychiatric care some respondents said that there was a formal policy at the workplace stating that professional interpreters were to be used, and not family members. This in contrast to somatic emergency care with its informal recommendation to use telephone interpreters or family members as quick and accessible alternatives. In ambulance care informal guidelines were also used, with the normal routine of using available family members. A respondent in ambulance care said that there is a written formal policy to use an interpreter but it was not adapted to their working conditions and thus could not be used. Booking an interpreter becomes an issue for health professionals in emergency care by self-established informal guidelines, a routine which the majority were pleased with.

*It works well, the policy, although I don’t have it on paper and … haven’t seen it … I think everyone at the emergency unit where I work has the policy established that there should be an interpreter in place or on the phone.* (R 26, Psychiatric EC)

Most of the respondents stated they had learned from older colleagues when and how to use a professional interpreter and a few from training or introduction at the workplace.

*Yes, it’s some kind of local tradition … transmitted both by older colleagues, when you see how they work. But … also from other occupational categories.* (R 13, Somatic EC)

Most health professionals expressed satisfaction with the informal procedures available and had no need for other guidelines. If guidelines were available it was perceived as contributing positively by increased used of interpreters and facilitation of equitable sharing. On the other hand, guidelines could restrict the use by being too detailed in their recommendations.

The majority of respondents knew how to order a professional interpreter, which was done from one or two interpreter agencies that the workplace had agreements with. Booking was done through a written document describing the process and was performed by nurses, nurse assistants, and/or care administrators. Physicians, managers, and staff in the ambulance service said they were never involved in booking a professional interpreter and did not know how to do it. No restrictions in terms of costs for the order were perceived to hinder the use of a professional interpreter; instead the needs of the patient determined the use.

*It is the responsible nurse, mostly … the nurse who does it … who has a memo saying this is what we do when we order an interpreter… But it’s more a matter of how you go about it, what number to call and what the customer code number… Interpreter service, I think it’s called.* (R 4, Somatic EC)

Some managers in emergency care described being aware of issues concerning interpreter use when staff experienced difficulties, or they handled the billing or issues of patient safety.

*I come into contact with it in that I pay the bills for interpreters … sometimes when the staff have difficulties … and discuss how we should act and think.* (R 11, Somatic EC)

#### Interpreters are used in situations with communication deficiencies in somatic and psychiatric emergency care

Booking of a professional interpreter was determined by health professionals themselves, identifying in the encounter with the patient the need for interpretation to be able to understand each other. Use of a professional interpreter was most frequently related to situations needing information exchange about the patient’s health status, e.g. assessment of the patient’s acute condition by physicians and obtaining an anamnesis, referrals to other institutions, exploration of symptoms, information about treatment or discharge from the emergency unit.

R 1:1: *… if they don’t speak any Swedish but just shake their heads and … don’t understand … then you have to … order a (professional) interpreter. Not when they get by in Swedish, then you don’t always order one … if the patient himself says that he wants an interpreter … it … oh … can surely be questioned from an ethical point of view what is right and from a patient safety perspective also maybe a (professional) interpreter ought to be ordered more often.* (Somatic EC)

A few respondents stated that interpreters are needed in all care settings for correct exchange of information. Many said that patients who need a professional interpreter usually have to wait longer, which may cause worry for the patient, or the patient might cancel the visit and refrain from care. On the other hand, a few described how the visit might go faster using a professional interpreter.

Conversation through a professional interpreter was perceived as often becoming technical and impractical, and the social chatter intended to relax the person was excluded. Further, there was less support for the patient with lack of emotional processing, and the patient gets more compressed information and lacks the opportunity to ask follow-up questions and become an active participant in healthcare. The health care staff say that the entire health care situation deteriorates because caring encounters are based on using language. Many respondents said that the relationship between the caregiver and the patient was negatively influenced when an unknown person in ordinary clothes was present in the room.

*That we can’t find a suitable interpreter or suitable dialect. Then care can be delayed … it takes more resources that we for example keep the patient on a bed for observation* (R 24, Psychiatric EC)

*It’s not a good caring situation. There will be no treatment or care… It feels frustrating that you can’t give them … because there is a lot of in conversation in our treatment, language is very important in caring.* (R 29, Psychiatric EC)

#### Professional interpreters are not used in ambulance care or in urgent situations

In care delivered in the ambulance or in the home, there is no time to get a professional interpreter in the right language due to short, fast, and unpredictable situations; instead family members or neighbors were used.

*… for patient contact in the home, but … we seldom use (professional) interpreters. The times we try to get hold of a (professional) interpreter it’s through SOS alarm and I find it difficult to get the help when we need it. Mostly when we’re out in contact with patients it’s so urgent that often you don’t have time to wait … you have to solve the situation then by trying to make yourself understood anyway … we seldom use that way but it’s not allowed to take more than five minutes at most.* (R 22, Ambulance care)

#### Professional interpreters are not used if body language can be used for communication

Professional interpreters are not used in short encounters in healthcare or in nursing care, e.g. meal situations or assessment of pain. Instead body language, family members, or bilingual colleagues are used by the nurses for initial assessment of the patient to sort out the cause of the visit or on admission to the emergency unit.

*If a patient comes in an emergent situation it’s the nurse who meets the patient and then we try with body language, English, Swedish, Google Translate, relatives, to get information about what the acute need is. The problem and the reason why they are here.* (R 25, Psychiatric EC)

*… check vital parameters … point to where it hurts and show with facial expressions…* (R 5) (Somatic EC)

In urgent situations or in case of severely ill patients or with lowered consciousness the patient’s status was assessed by bodily parameters and observations and thus professional interpreters were not used.

*see that there is something … very serious so maybe you start to look at all the vital parameters and ECG … before you can … start asking questions … to see if there is something super-super-fast that needs to be done.* (R 3, Somatic EC)

### The use of a professional interpreter depends on the interpreter’s linguistic skills, personal qualities, professional approach, and organizational aspects

#### The professional interpreter’s linguistic competence, positive personal qualities, and professional approach facilitates work when communication is deficient

Interpretation was described by the respondents to be of good quality when the professional interpreter had good language competence, in Swedish, the native language, and health care terminology, and paid attention to cultural expressions, translated word-for-word with a flow, and had good conversation technology, was neutral, could keep the code of confidentiality, and control him/herself in the emergency situation. Another influencing factor was the professional interpreter’s ability to establish a trustful and empathetic relationship, best done with a professional interpreter on the spot, which also gave the possibility to observe body language.

*… it’s fundamental in the interpreter’s profession that you know the language really well. Both Swedish and the other language. So that you get a clear picture and can translate correctly … important that we get what the patient is and not an interpretation of what the patient says.* (R 13, Somatic EC)

Sometimes even the patient’s own feelings could be seen as a barrier, e.g. when feeling unwell and being completely silent, or when emotions like fear and anger hinder the ability to listen.

The majority of health care staff in psychiatric and somatic emergency care have a positive attitude to the use of professional interpreters at the workplace, but some do not have any expectations or do not discuss the use of interpreters, particularly in the ambulance units where professional interpreters are rarely used.

#### Professional interpreter perceived as positive and as a tool facilitating communication

The professional interpreter is described by many as a tool overcoming communication deficiencies and as a solution to the language problem

*I think … everyone thinks it’s positive that it exists… There’s no one who thinks it’s troublesome, no. I don’t know, I haven’t heard anything at least. Sure, some might think it’s hard to talk by phone… It’s a bit uncomfortable to talk to someone who isn’t there for you to see.* (R 4, Somatic EC)

#### Professional interpreter perceived positively or negatively when organizational aspects (time, environment, and technical equipment and interpreter languages) of the use of interpreters work or not

When the professional interpreter is available on time and stays as long as needed, and when the technical equipment functions well and the interpretation occurs in an undisturbed environment, health professionals felt that these organizational and practical aspects functioned and the interpreting situation was good. In the case of women seeking help for gynecological problems, some found it beneficial to have a female professional interpreter.

When technical problems occur, or there is lack of space making it difficult to maintain confidentiality, or when the professional interpreter who has been ordered speaks the wrong language, the interpretation situation does not function.

*There are all the peripheral factors. That you have to be sitting in a good place, where there is plenty of space, that you have allocated time for the interpretation, and that you can be undisturbed.* (R 14, Somatic EC)

### Recommendations to improve the use of interpreting services in emergency care

#### Developing the procedure for prompt access to professional interpreters in the workplace

The healthcare professionals indicate that the use of professional interpreters would be facilitated and improved with fast access to professional interpreters by a hotline phone round the clock, and many wished for better access to the most common languages as well as to all languages.

*… that there was some kind of quick track to the interpreter service … it goes really fast in many … in many cases they call them back at once when you have the phone … for the most common languages they could guarantee an interpreter in five minutes.* (R 8:3, Ambulance care)

Some discussed how better technology and technical solutions could improve professional interpreter use and the organization of interpreting would be facilitated if the administrative staff helped to book a professional interpreter. For health professionals in ambulance care, it would help if the SOS alarm staff booked a professional interpreter in advance, so that the interpreter was available from the first contact onwards, and all types of professional interpretation were thought to help to make it better for patients and health professionals in ambulance care

*Good instrument when interpreting, either good telephones with good sound but also perhaps a video link … as you talk to someone over the telephone you just as easily could be able to look the interpreter the face … a face that also sees the patient.* (R 3, Somatic EC)

*Some staff believe a “joint policy” on (professional) interpreter use could lead to improvement, provided that the policy does not regulate interpreter use for certain care situations.* (R 30, Psychiatric EC)

#### Education of health professionals in using a professional interpreter, and the professional interpreter’s role in various care situations

Some said that the interpretation situation would improve if the patient’s perspective was considered more often, if a professional interpreter was used more frequently used during nursing care situations, if staff were trained in how to act and use a professional interpreter, and if the professional interpreter is trained to have a professional attitude and to adapt to the caring unit’s specific requirements.

*… what one can think about is the patient perspective on interpretation, that we’re probably not always so good at asking for their point of view.* (R 30, Psychiatric EC)

*If anything would make it easier for me it would be to have slightly clearer guidelines on how they are trained, what kind of degree of confidentiality they have, can you reach more people without adding your own values in an interpreter conversation at our workplace, it’s more that kind of information I would wish.* (R 25, Psychiatric EC)

## Discussion

This study explored language interpretation practices in multilingual emergency healthcare by studying different health professionals describing their actions when organizing language interpreting. Thus, comparisons with previous studies will only be partial. The main results showed that language interpretation services in emergency care are organized based on the patient’s health status, and the context of emergency care and access to the interpreter service in the organization determine the use. Bilingual healthcare staff and family members were used, but to a limited extent, in somatic emergency care, in contrast to psychiatric emergency care where the norm was to have professional interpreters present. In ambulance service professional interpreters were seldom used; instead persons available at the moment, such as family members, friends etc., were used, along with observation of body language. Booking of a professional interpreter was mainly based on informal, collectively constructed guidelines and routines at the workplace and sometimes on formal guidelines and different national laws, but knowledge of existing laws was limited. The ideal was a professional interpreter with high linguistic competence and a professional attitude, and organizational aspects such as appropriate time and technical and social environment. Finally, wishes for the future included the development of a better procedure for prompt access to professional interpreters at the workplace, regardless of organizational context, and education of interpreters and their users.

In this study, healthcare staff determined the use of professional interpreters in multicultural emergency care based on the patient’s health status, the kind of emergency care, and access to the interpreter service in the organization to assess as fast as possible the individual’s need of care, in contrast to multicultural elderly care where the use of professional interpreters was determined by medical consultations with physicians or for individual care planning activities [13]. Communication can be described as a multifaceted phenomenon and through communication the person’s identity is made visible and shows how the other person is perceived on the individual and the social level [26]. It also makes it possible for the individual to participate in society. Further, communication in an institutional context is asymmetrical when patients and healthcare staff do not have the same aim, knowledge, or resources [17]. This study found that communication through interpreters is a complex process that depends on several factors such as patients’ health status, context of emergency care, and access to interpreting service, but also on informal and formal guidelines governing the workplaces. However, in an emergency situation neither shared decision-making nor participation in communication might be possible due to the patient’s health status, e.g. being unconscious, having extreme pain, etc., and the urgency of the situation [16]. Thus, an asymmetrical power relation might need to be accepted temporarily to help the individual retain or regain health. Communication is central for caring, but in such situations it might not be fully possible to provide transcultural care where the individual’s needs and cultural beliefs are taken into account [16] and person-centered, safe, and equal care can be delivered in accordance with Swedish law [35, 36] and human rights [26] including the legal and health literacy [28]. Health professionals in this study described how they selected certain situations for interpreting, in contrast to care for people who speak the official language of the country. This can be explained as caring routines and might be helpful for a person in a stressful situation [21] but it can contribute to further development of unequal care [35, 36]. The findings emphasize the need for legal and health literacy and social justice to help reduce inequalities in accessing healthcare by empowering migrant patients to understand and critically engage in their healthcare [28]. Patients in need of an interpreter received less support in dealing with emotions, often had to wait longer, and had more compressed information and lack of opportunities to ask follow-up questions than native-born people, which is similar to findings from a review study concerning emergency healthcare which found that patients in need of an interpreter were less satisfied, received inferior care, had limited diagnosis and treatment, and fewer follow-ups [6]. An interesting finding in this study is that no one mentioned economic aspects of the booking or use of an interpreter. Thus, care in the studied area is focused on humanism rather than bureaucratic values [21].

In the present study the use of professional interpreters was related to the organizational context, with more frequent use in somatic and psychiatric emergency care than in the ambulance service, where professional interpreters were not used and instead those available at the time, e.g. relatives and bilingual staff, were used to a higher extent. This is in contrast to multicultural elderly care where no dissimilarities were found between different sectors such as community home elderly care, nursing homes, and nursing homes for dementia patients [13]. This is possibly related to organizational routines [18, 19] and organizational cultural competence [16]. An important aspect here is the characteristics of the environment and context in which care is delivered. Ambulance service may differ from somatic and psychiatric emergency care particularly due to the limited space, but also limited timeframes in which care is given and can thus act as barriers to access, availability, and use of interpreters. Thus, use of interpreters needs to be adapted to the environment and context. In the present investigation, however, interpreter use was determined by individual healthcare staff based on collectively constructed informal guidelines at the workplace and sometimes discussed with workmates; it thus mainly covered only the individual and interpersonal levels of organizational routines described in the theoretical framework [18, 19]. There is a need for development of the three other levels: the organization, the institution, and the environment. Unclear routines affect how health professionals deliver care, and absence of guidelines and common objectives entails a risk that unconscious behaviors based on staff’s own standards and attitudes might negatively affect healthcare encounters [22] with migrants. Routines enable coordination, ensure some stability of behavior, and when tasks are routinized they can be performed subconsciously, thereby economizing on limited cognitive energy [40]. Improved knowledge and development of strong routines can shape common practice that improves the delivery of service [18] such as interpreters.

As in previous studies [13, 37], it was considered preferable to get an interpreter with high linguistic competence and a professional attitude, and satisfactory organizational aspects such as appropriate time and technical and social environment. In emergency care the interaction with an interpreter showing trust, confidence and empathy is important and fundamental for the quality of the conversation, and the interpreter needs to learn how to contribute to the alliance in healthcare and thus also learn about the context in which healthcare is delivered [41]. This needs to be developed in existing guidelines for authorized interpreters in Sweden, which mainly focus on interpretation technique, translating correctly and neutrally [42] and not on the interpreter’s professional attitudes or organizational routines. Knowledge about existing national laws and policies for using and booking interpreters in Sweden differed but was in general limited among the respondents, and the use of interpreters was seldom on the agenda for managerial staff in emergency care. However, no national guidelines exist with the exception of those mentioned above formulated by Kammarkollegiet [42] and the Management Act [11], stating the right to get an interpreter in contact with public authorities, and the Public Procurement Act [34] regulating what companies interpreter service can be procured from, and so this finding is not surprising. Sweden, like many other European countries, has become a multicultural society with a high influx of migrants in recent years, with an increasing demand for interpreters [43], particularly in healthcare, which is unmet due to lack of interpreters [44]. Political interventions are needed to solve this. There is an obvious knowledge gap to fill here.

### Limitations of the study

It might be seen as a limitation that a mix of individual interviews and focus-group interviews was used for data collection. However, it has been claimed that the same information can be reached by focus-group interviews as by individual interviews, although more time is needed for the individual interviews [45]. On the other hand, it is a strength to combine different methods for data collection, and this strengthens the findings [30]. Not including patients’ and interpreters’ views in the study could be seen as a limitation but was impossible due to the available resources for this investigation; this needs to be further investigated.

The focus-group interviews were held without an observer present, which can be seen as a limitation [31] but as a small group design [46] was applied, the interviewers had the same professional background as the respondents, and were also familiar with leading and documenting interactions in groups, in both research and education, the influence of this is considered negligible [31].

The main limitation of a qualitative study is that the findings cannot be generalized or explain cause-effect relationships [30, 31], but the main aim of this study was to explore reality in order to arrive at a deeper understanding of the phenomenon, and the findings are transferable to other contexts similar in characteristics and can contribute new knowledge in developing similar organizations.

## Conclusions

In conclusion, the data seem to show that health professionals act as gatekeepers for migrants’ access to interpreters and fail to apply Swedish laws. The use of professional interpreters in multicultural emergency care was determined by health professionals based on the patient’s actual health status, and they did whatever was possible to deliver fast and individualized care as needed and based on humanistic values. However, there are shortcomings in the institutions’ organizational routines that need to be rectified concerning organizational, institutional, and environmental factors and the importance of transcultural awareness to achieve the aim of person-centered and equal health care. The main task is to develop clear formal guidelines for the use of interpreters at the workplaces, including the procedure for the use depending on the patient’s desire, health status and type of emergency healthcare, but also to fulfill the health professionals’ wishes for future development of prompt access to interpreters, education of interpreters and of users of interpreters as regards how to perform interpretation.
